# Cohort Profile: The Study of Cognition, Adolescents and Mobile Phones (SCAMP)

**DOI:** 10.1093/ije/dyy192

**Published:** 2018-10-15

**Authors:** Mireille B Toledano, Julian Mutz, Martin Röösli, Michael S C Thomas, Iroise Dumontheil, Paul Elliott

**Affiliations:** 1 MRC-PHE Centre for Environment and Health, Department of Epidemiology and Biostatistics, School of Public Health, Faculty of Medicine, Imperial College London, London, UK; 2 National Institute for Health Research Health Protection Research Unit in Health Impact of Environmental Hazards at King's College London, a partnership with Public Health England, and collaboration with Imperial College London, London, UK; 3 Social, Genetic and Developmental Psychiatry Centre, Institute of Psychiatry, Psychology and Neuroscience, King's College London, London, UK; 4 Department of Epidemiology and Public Health, Swiss Tropical and Public Health Institute, Basel, Switzerland; 5 University of Basel, Basel, Switzerland; 6 Centre for Educational Neuroscience, Department of Psychological Sciences, Birkbeck, University of London, London, UK

## Why was the cohort set up?

The Study of Cognition, Adolescents and Mobile Phones (SCAMP) is a prospective secondary school-based cohort study established to investigate whether use of mobile phones and other wireless devices that emit radio-frequency electromagnetic fields (RF-EMF) is associated with cognitive, behavioural, educational, physical and mental health outcomes during adolescence. Specifically, the principal aim is to discern whether any observed associations may be due to: (i) RF-EMF exposure from mobile phones; (ii) a combination of various RF-EMF sources (e.g. digital enhanced cordless technology phones or wireless internet); or (iii) other behavioural reasons associated with technology use for communication and entertainment, irrespective of exposure to RF-EMF.

Mobile phone use is widespread amongst children and adolescents, with market research suggesting that 43% of 8-11-year-olds and 86% of 12-15-year-olds in the UK own a mobile phone.^1^ In 2000, the UK government-commissioned *Stewart Report* highlighted that children and adolescents may be ‘more vulnerable [to potential adverse health effects resulting from mobile phones] because of their developing nervous system, the greater absorption of energy in the tissues of the head and a longer lifetime of exposure’.^2^ This concern has been echoed in other publications,^3^^,^^4^ and the possible health effects of RF-EMF have since been extensively reviewed. Although governments, non-governmental organizations and professional bodies have put forward recommendations and implemented policies to limit children’s and adolescent’s RF-EMF exposure,^5^ scientific uncertainty and public concern remain about the potential adverse health effects of RF-EMF from mobile phone use.

The 2010 World Health Organization (WHO) research agenda for RF fields ranked prospective cohort studies of children and adolescents as the highest priority research need.^6^ SCAMP was designed to address gaps in and limitations of the current knowledge base by: (i) focusing on adolescence, when personal mobile phone use becomes predominant; (ii) prospectively collecting self-reported information on mobile phone use together with traffic data from network operators; and (iii) assessing longitudinally cognitive and behavioural development in relation to use of mobile phones and other wireless devices.

This research will help to inform UK and global health policy on adolescents’ mobile phone use. Additionally, the collection of data on other environmental exposures (e.g. air pollution, noise, green space use) will generate a rich dataset beyond RF-EMF exposures, which will allow for research on a wide range of other environmental and health issues in this important age group.

The North West-Haydock Research Ethics Committee approved the SCAMP study protocol and subsequent amendments. The study is conducted in accordance with the Declaration of Helsinki (1964 and later revisions).

## Who is in the cohort?

**Figure 1.**
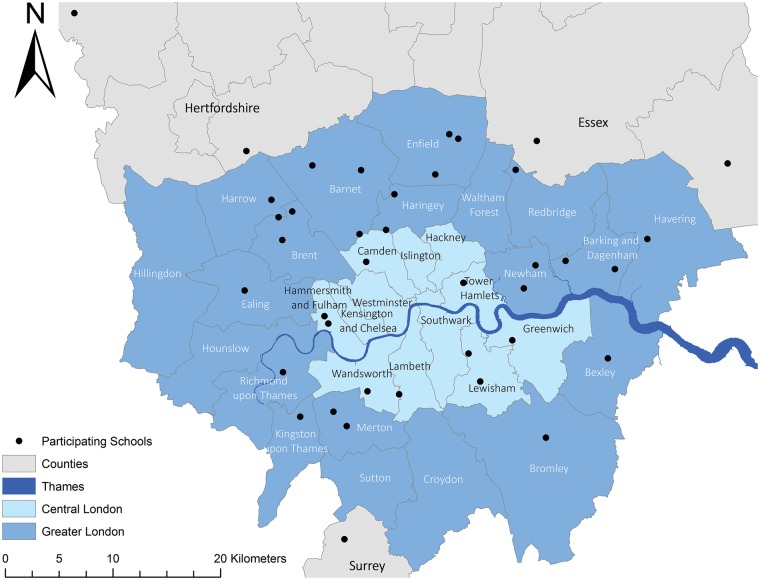
Map of schools participating in SCAMP.

The SCAMP cohort consists of *N *=* *6905 pupils. Assessments were undertaken in Year 7 (first year) of 39 secondary schools (26 state, 13 independent) in and around Greater London (Figure 1). Eligible schools were selected from the Department of Education’s register of educational establishments (EduBase) and from the January 2012 school census.^7^^,^^8^ Both datasets include information on the type of school (e.g. independent school), pupil characteristics (e.g. sex), geographical location and pupil headcounts by school year or age.

To select schools with pupils in the target age range (11-15-year-olds), any school classified as a primary, infant, junior, or middle school or with a statutory minimum age of 12 years was excluded. Any school classified as a special school, pupil referral unit or secure unit was also excluded as not representative of the general school-age population. Schools were included if they had a total Year 7 headcount of *N *>200 or *N *>50 pupils, for state and independent schools respectively.

**Figure 2.**
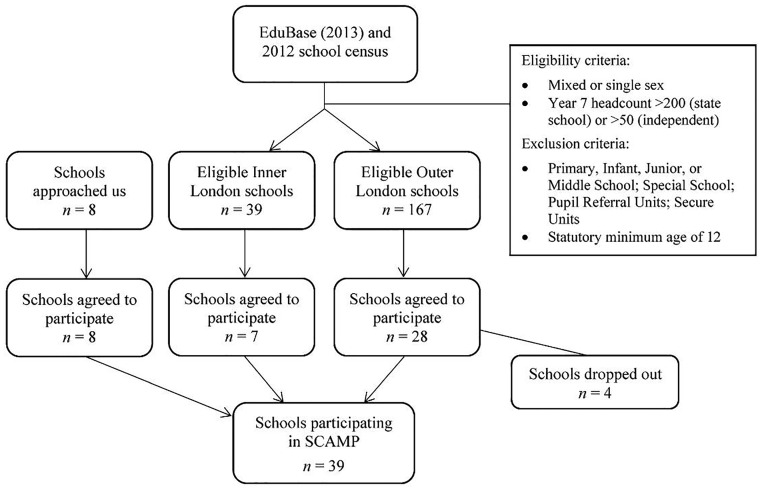
Flowchart of school recruitment for SCAMP (baseline).

167 eligible schools in Outer London were identified and mailed invitations to take part in SCAMP; 28 schools (19 state, nine independent) initially agreed to participate. An additional 39 schools in Inner London were invited to participate. Seven agreed to take part (three state, four independent). Through word of mouth and communication with schools and headteacher associations, another eight schools that met our eligibility criteria approached us to participate in SCAMP and were subsequently included in the cohort. Four schools dropped out of the study before data collection due to logistical or technical issues, after having initially agreed to participate (however, *n *=* *13 pupils from two of these schools decided to participate individually at Imperial College London and were included in our analyses). Figure 2 provides an overview of the school recruitment process.

All parents of Year 7 pupils (aged 11–12 years) from participating schools received information packs about the study before the school assessments took place. Participation in SCAMP is voluntary and if parents did not want their child to take part in the assessment, they were asked to contact the research team (opt-out). Pupils can also decide not to participate in any part of the study at any point in time.

Of the *N *=* *7375 (according to school register data) Year 7 pupils at participating schools, *n *=* *6616 (89.71%) took part in the school-based computer assessment at baseline between November 2014 and July 2016. Of this non-participation, 14.62% (*n *=* *111) is due to parental opt-out; the remaining 85.38% (*n *=* *648) can be accounted for by absentees, non-assents by participants, withdrawals, technical issues or miscellaneous reasons.

## How often have they been followed up?

### Direct follow-up with adolescents

From November 2016 through July 2018, adolescents undertook a follow-up computerized assessment when they are in Year 9/10 (13–15 years old). As of July 2018, the estimated rate of attrition from the baseline computerized assessment was 24% (eight schools; *n* ∼ 1593 adolescents).

### Passive follow-up via record linkage

Parental consent is requested for linkage of adolescents’ school assessment data to routinely collected data, including health and educational records as well as mobile phone traffic data from network operators. As of July 2018 we have received parental consent for data linkage for *n *=* *1318 (20%) of the pupils in our cohort.

## What has been measured?

Table 1 provides an overview of the data that are being collected as part of the SCAMP computer assessment and the online questionnaires. Briefly, Year 7 pupils complete a series of questionnaires and a cognitive test battery on eight cognitive domains (non-verbal fluid intelligence, speech processing, cognitive flexibility, sustained attention, inhibition, working memory, visual attention and mental rotation), which are both embedded in a smart client software (Psytools, Delosis Ltd). The assessments take place under exam conditions during regular school time for a duration of approximately 60 min, with at least one researcher supervising each session. Approximately 2 years later, when pupils are in Year 9/10, the assessments are repeated under similar conditions. The school-based component of SCAMP is complemented by consent/assent registration and questionnaires that are accessible online and can be completed in any environment by both parents and pupils.

**Table 1.** SCAMP data collection (school assessment and online questionnaire)

	School assessment		Online questionnaire	
	Baseline	Follow-up	Child	Parent
Cognitive assessment				
Non-verbal fluid intelligence: Cattell Culture Fair Test^9^	✓	✓		
Speech processing: Speech-in-Noise Task^10^	✓	✓		
Cognitive flexibility/task switching: Trail Making Test^11^^,^^12^	✓	✓		
Sustained attention: AX-Continuous Performance Test^13^	✓	✓		
Inhibition Find-the-Phone Task;^14^ AX-Continuous Performance Task^13^	✓	✓		
Working memory: Backwards Digit Span Task;^15^ Find-the-Phone Task;^14^ Dot Matrix Task^15^^,^^16^	✓	✓		
Visual attention: Enumeration Task^17^^,^^18^	✓	✓		
Mental rotation: Mental Rotation Task^19^	✓	✓		
Questionnaires				
Mobile phone				
Current mobile phone ownership	✓	✓	✓	
Mobile phone details e.g. make, smartphone	✓	✓		
Age first using a mobile phone	✓	✓		✓
Age regularly using mobile phones				✓
Use of other people’s mobile phones	✓	✓		✓
Details on callers e.g. parents, friends	✓	✓		
Frequency/duration of calls weekday, weekend	✓	✓		
Location of mobile phone when carrying/talking	✓	✓		
Use of hands-free services	✓	✓		
Parental encouragement to use hands-free services				✓
Mobile internet use including proportion using WiFi	✓	✓		
Messaging frequency text and instant messages	✓	✓		
VoIP calls including type of connection and device	✓	✓		✓
Long calls including somatic effects	✓	✓		
Restricting mobile phone use	✓	✓		✓
Hours of mobile phone use allowed daily				✓
Type of contract and expenses/PAYG amount				✓
Problematic mobile phone use behaviour			✓	
Night-time mobile phone use; device use before sleep	✓	✓		
Cordless phone				
Duration of calls weekday, weekend	✓	✓		
Location of base station				✓
Time phone docked into base station				✓
Device use at school				
Desktop computer	✓	✓		
Laptop	✓	✓		
Tablet	✓	✓		
Device use outside school				
Desktop computer	✓	✓		
Laptop	✓	✓		
Tablet/ebook reader	✓	✓		
Media player	✓	✓		
Gaming console portable/nonportable	✓	✓		
Smart TV	✓	✓		
Video games				
Frequency of play including type of games	✓	✓		
Playing alone or in group		✓		
Use of other technologies				
E-mail	✓	✓		
TV	✓	✓		
Internet	✓	✓		✓
Parental restriction on daily internet use				✓
Social networking	✓	✓		
Music headphones, speaker	✓	✓		
WiFi at home including router location, night-time switching off				✓
Number of wireless devices in household				✓
Smart house				✓
Smart meter				✓
Health and well-being				
Health-related quality of life: KIDSCREEN-10^20^	✓	✓		
Sleep length, latency, quality, disturbance	✓	✓		
Hearing and tinnitus	✓	✓		✓
Headaches	✓	✓		
Disabilities, illness or medical condition				✓
Prescriptions, medications, therapy				✓
Head trauma, brain surgery, exposure to radiation				✓
Suffered electric shocks				✓
Learning disabilities and/or other special education needs including attention-deficit hyperactivity disorder				✓
Familial special education needs				✓
Giftedness				✓
Symptoms of depression: PHQ-9^21^		✓		
Symptoms of anxiety: GAD-7^22^		✓		
Cyber bullying		✓		
Body image			✓	
Puberty			✓	
Life-changing events	✓	✓		
Pregnancy and child development				
First child, number of siblings				✓
Use of wireless devices during pregnancy				✓
Smoking, alcohol and caffeine consumption during pregnancy				✓
Dietary restrictions during pregnancy				✓
Exposure to chemicals during pregnancy				✓
Born prematurely, complications during pregnancy or at birth				✓
Birthweight				✓
Breastfeeding behaviour				✓
Behaviour				
Emotional symptoms, conduct problems, hyperactivity or inattention, peer relationship problems, prosocial behaviour: SDQ^23^	✓	✓		✓
Self-efficacy			✓	
Domain-specific impulsivity: DSIS-C^24^			✓	
Leisure activities				✓
Musical instruments			✓	
Sport and physical activity		✓	✓	
Diet	✓	✓	✓	✓
Eating habits and factors affecting food intake				✓
Smoking, alcohol, and cannabis consumption	✓	✓		
Sociodemographics				
Age	✓	✓		
Sex	✓	✓		
Religion	✓	✓		
Handedness				✓
Height	✓	✓		✓
Weight	✓	✓		✓
Parental height				✓
Parental weight				✓
Parental education	✓	✓		✓
Parental occupation	✓	✓		✓
Family allowances and income				✓
Free school meals				✓
Household and family structure				✓
Own bedroom/disturbance by roommates	✓	✓		✓
Home address	✓	✓		
English first language	✓	✓		✓
Language talking to parents	✓	✓		✓
Environmental factors				
Smoking in home environment	✓	✓		✓
Travelling to school including living near busy road	✓	✓		
Noise exposure indoor and outdoor			✓	
Use of green and blue spaces according to seasons	✓	✓		
Typical activities in green and blue spaces		✓		
Damp or mould in home environment				✓
Cooking, windows, and ventilation at home				✓

Table shows data that are collected during the SCAMP computer-based school assessment and which are included in the optional online questionnaires.

PHQ-9, Patient Health Questionnaire;^21^ GAD-7, Generalised Anxiety Disorder Assessment;^22^ SDQ, Strengths and Difficulties Questionnaire;^23^ DSIS-C, Domain-Specific Impulsivity Scale for Children.^24^

### Data linkage

For those adolescents for whom we have received parental consent, approval has been obtained for linkage to health records. This will allow long-term health follow-up through hospital episode statistics (HES) and birth, mortality and cancer registration records, via NHS Digital or the Office for National Statistics. Linkage to adolescents’ data on mobile phone calls, texting and data downloads is undertaken annually via the mobile network operators (Table 2).

**Table 2.** SCAMP enhancements data collection

**Personal monitoring**	
RF-EMF	
16 frequency bands (87.5–5875 MHz) incl. GPS data for 48-72 h (ExpoM-RF)	
Smart phone activity diary including GPS data for 48–72 h	
Noise	
Measured	Modelled
Fixed-site monitor assessments of hourly LAeq and Lmax (home and at school: indoors and outdoors)	Outdoor noise from different transport sources for each home address and school location
	Road traffic noise for each building (TRANEX^(^25^)^)
	Rail noise data for each address (ICL)
	Airport noise data for London Heathrow and London City airport (ICL)
Air pollution	
Measured	Modelled
PM_2.5_, PM_10_, NO_X_ (NO and NO_2_), O_3_ and particle number concentrations (home and at school: indoors and outdoors)	NO_X_ (NO and NO_2_), O_3_, PM_2.5_, PM_10_ (separated into primary tailpipe and non-tailpipe sources) (LHEM^26^)
	Average exposure for different seasons, weekend and weekday, as well as mobility (in-vehicle, train, cycling)
Non-invasive biological samples (first morning void urine and saliva samples)	
Exposure biomarkers	
Environmental tobacco smoke	
Brake wear (Cu, Sb, Ba)	
Tyre wear (Zn)	
Resuspension of road dust (Al, Ca)	
Mechanical abrasion from the engine (Fe, Mo, Mn)	
Tailpipe markers indicative of oil/fuel combustion (Cr, Ni, V, As)	
Other biomarkers	
Pubertal status	
Stress (cortisol)	
Genotype (saliva sample)	
DNA sample (ORAgene^®^)	
Android phone data (XmobiSense^27^)	
Frequency/duration of mobile phone calls	
Use of speakerphone and hands-free services	
Volume of data uploads/downloads (WLAN and mobile network)	
Type of network	
Laterality of phone use	
Internet/VoIP calls (WLAN and mobile network)	
iOS phone data	
Call time	
Mobile data	
Dietary app (MyFood24^28^)	
Nutritional intake for 24 h	
**Bio-Zone**	
Non-invasive biological samples	
Exposure biomarkers (urine and saliva samples)	
Environmental tobacco smoke	
Brake wear (Cu, Sb, Ba)	
Tyre wear (Zn)	
Resuspension of road dust (Al, Ca)	
Mechanical abrasion from the engine (Fe, Mo, Mn)	
Tailpipe markers indicative of oil/fuel combustion (Cr, Ni, V, As)	
Other biomarkers (urine and saliva samples)	
Pubertal status	
Stress (cortisol)	
Genotype (saliva sample)	
DNA sample (ORAgene^®^)	
Anthropometric measurements	
Height (cm)	
Weight (kg)	
Waist circumference (cm)	
Grip and pinch strength (kg)	
Spirometry	
Forced vital capacity (VFC)	
Forced expiratory volume in 1 s (FEV1)	
**Data linkage**	
Mobile network operator data	
Frequency/duration of mobile phone calls	
Numbers of SMS	
Volume of internet traffic data	
Educational achievement data	
School exam results	
Key Stage 2 and 3 results	
Cognitive Abilities Test (CAT) results	
Information from National Pupil Database	
Information about Special Educational Needs	
Health data	
HES admitted patient care	
HES critical care	
HES outpatients	
HES accident and emergency	
Diagnostic imaging dataset	
ONS Mortality data	
Birth records	
Cancer registration data	
Primary care data (where available)	

Table shows data that are collected as part of SCAMP’s personal monitoring and Bio-Zone enhancements as well as data requested following parental consent.

RF-EMF, radio-frequency electromagnetic fields; MHz, megahertz; GPS, global positioning system; TRANEX, traffic noise exposure model;^25^ ICL, Imperial College London; LHEM, London hybrid exposure model;^26^ HES, hospital episode statistics; ONS, Office for National Statistics.

### Biological samples collection (SCAMP Bio-Zone)

To provide additional information on covariates (e.g. puberty, stress, exposure to environmental tobacco smoke, genotype), non-invasive biological samples (urine and saliva) and anthropometric measurements (height, weight, waist circumference, grip and pinch strength) are being collected in 12/39 schools (Table 2). All schools were invited to participate in SCAMP Bio-Zone. The pupils taking part in SCAMP Bio-Zone (*n *=* *2270) are also in the main cohort, unless they were absent during the day or at the time of the computer assessment. This resulted in *n *=* *289 adolescents who participated in Bio-Zone but who did not complete the computer assessment.

### Personal and home environmental exposure monitoring

A subset (*n* ∼ 200) of the main cohort participates in a personal exposure monitoring study that started after the baseline data collection. The aims of this study are to: (i) gain an in-depth understanding of personal and home exposures to RF-EMF, noise and air pollution that is representative of real-life situations; (ii) differentiate exposure to RF-EMF from mobile phone use and from other sources; (iii) enhance and validate the exposure assessment for the main cohort; and (iv) assess in detail exposure-relevant behaviour and characteristics. All pupils receive an invitation to participate in the personal and home environmental exposure monitoring study after they have participated in the school assessment, through an information pack that is either sent to the parental home address or handed out to the adolescents at school.

Briefly, adolescents carry a portable RF-EMF measurement device (ExpoM-RF) for a duration of 48–72 h to assess their personal exposure to 16 frequency bands (87.5–5875 MHz). They also complete a time-activity diary on a study smartphone that is in flight mode. The ExpoM-RF records global positioning system (GPS) data for the entire duration of the personal monitoring study, and the study smartphone records GPS data each time an activity is being logged in the diary. Exposure to air pollution and noise at home is measured throughout the same period using fixed-site indoor and outdoor monitors. Adolescents also complete a paper-based self-report questionnaire about exposure-relevant factors and mobile phone use (Supplementary Table 1, available as Supplementary data at *IJE* online) and provide first morning-void urine and saliva samples on the last day of the measurement period.

Finally, Android users are encouraged to provide further in-depth data via the smartphone application XmobiSense^27^ (this is unavailable for iPhones, which use the iOS operating system). The application records the number of phone calls, time spent talking on the phone, use of speaker phone or other hands-free services, side of head (laterality), amount of data uploaded/downloaded and type of network [e.g. second- or third-generation (2G or 3G)]. The main advantage of XmobiSense is that data transfers (uploads/downloads) over the mobile phone network and over the wireless local area network (W–LAN) network are recorded separately. Moreover, for internet/voice-over internet protocol (VoIP) calls (e.g. Skype), XmobiSense can differentiate those made through the mobile phone network from those made via W–LAN (Table 2). As only relatively few pupils use Android phones, individual phone usage data are also downloaded directly from iOS phones where possible.

## What has it found? Key findings and key publications from the baseline study

Baseline data collection was completed in July 2016. Table 3 provides an overview of the baseline sociodemographic characteristics of the SCAMP cohort that took part in the computer-based school assessment. For comparative purposes, Table 3 also shows these characteristics for the target Greater London school population from census data. Characteristics of mobile phone use at baseline are presented in Table 4. The sociodemographic characteristics of the SCAMP Bio-Zone cohort at baseline are presented in Supplementary Table 2, available as Supplementary data at *IJE* online.

**Table 3.** Baseline sociodemographic characteristics of the SCAMP cohort^a^

	Target population^b^	Overall		Male		Female	
	–	(*N* = 6616)		(*n* = 3147, 47.57%)		(*n* = 3469, 52.43%)	
	Range	Median	IQR	Median	IQR	Median	IQR
Age (years)^c^	11-12	12.07	11.79-12.34	12.09	11.82-12.37	12.04	11.76-12.31
Ethnicity	**%**^d^	***n***	**%**	***n***	**%**	***n***	**%**
White	41.08	2669	40.34	1310	41.63	1359	39.18
Black	21.33	972	14.69	472	15.00	500	14.41
Asian	21.23	1670	25.24	745	23.67	925	26.66
Mixed	8.70	683	10.32	335	10.65	348	10.03
Other/not interpretable	5.60	373	5.64	172	5.47	201	5.79
Missing	2.07	249	3.76	113	3.59	136	3.92
Socioeconomic classification							
Managerial/professional occupations	39.75	3270	49.43	1554	49.38	1716	49.47
Intermediate occupations	13.70	484	7.32	203	6.45	281	8.10
Small employers and own-account workers	10.43	910	13.75	462	14.68	448	12.91
Lower supervisory and technical occupations	5.81	272	4.11	132	4.19	140	4.04
Semi-routine/routine occupations	20.82	693	10.47	314	9.98	379	10.93
Missing/not interpretable	9.49	987	14.93	482	15.32	505	14.56
Type of school							
State	76.78	5141	77.71	2522	80.14	2619	75.50
Independent	23.22	1475	22.29	625	19.86	850	24.50

The socioeconomic classification is based on the highest National Statistics Socioeconomic Classification (NS-SEC) level (five-group version) of either parent.

a Data based on participants who took part in the computer-based assessment.

b Data on ethnicity and type of school of target population is based on the January 2015 School Census [www.gov.uk/government/statistics/schools-pupils-and-their-characteristics-january-2015]; data on socioeconomic classification is based on the 2011 Census: NS-SEC in London [https://data.london.gov.uk/dataset/ns-sec-report-data].

c *Data on age missing for* n = 19 participants.

d Percentages for ethnicity in target population available for state-funded secondary schools only.

**Table 4.** Baseline mobile phone use characteristics of the SCAMP cohort^a^

	Weekday		Weekend			Weekday		Weekend	
	*n*	%	*n*	%		*n*	%	*n*	%
Call frequency					Call duration/day				
Never	448	6.77	807	12.20	0 min	524	7.92	812	12.27
Few/month	1198	18.11	1224	18.50	1–5 min	2557	38.65	1792	27.09
Few/week	1410	21.31	1032	15.60	6–15 min	1237	18.70	1243	18.79
∼1/day	823	12.44	753	11.38	16–30 min	525	7.94	682	10.31
2–5/day	1068	16.14	898	13.57	31–59 min	263	3.98	372	5.62
6–10/day	328	4.96	411	6.21	1–2 h	209	3.16	293	4.43
11–20/day	115	1.74	209	3.16	≥3 h	175	2.65	296	4.47
≥21/day	100	1.51	156	2.36					
Missing	1126	17.02	1126	17.02	Missing	1126	17.02	1126	17.02
SMS texts					Instant messages				
None	1023	15.46	1164	17.59	None	915	13.83	865	13.07
1–5/day	1978	29.90	1513	22.87	1–5/day	1117	16.88	905	13.68
6–10/day	983	14.86	900	13.60	6–10/day	969	14.65	819	12.38
11–40/day	813	12.29	897	13.56	11–40/day	1001	15.13	1008	15.24
41–70/day	287	4.34	393	5.94	41–70/day	465	7.03	602	9.10
71–100/day	168	2.54	253	3.82	71–100/day	288	4.35	369	5.58
≥101/day	234	3.54	366	5.53	≥101/day	360	5.44	547	8.27
Missing	1130	17.08	1130	17.08	Missing	1501	22.69	1501	22.69

Instant messages include e.g. Whatsapp, iMessage, Instagram Direct, Snapchat.

a Data based on participants who took part in the computer-based assessment.

### Mobile phone ownership

With respect to the cohort participating in the computerized assessment, *n *=* *125 (1.89%) reported that they never owned a mobile phone and *n *=* *423 (6.39%) indicated that they used to own a mobile phone but do not currently own one. Most pupils [*n *=* *5492 (83.01%)], reported that they owned a mobile phone at the date of the baseline school assessment,^29^ and there was no difference between male and female adolescents, *P *=* *0.095. However, mobile phone ownership differed according to adolescents’ age, ethnicity, parental socioeconomic classification and type of school (*P *<0.001, Mann-Whitney *U* or *χ*^2^ test).

### Variables associated with mobile phone ownership

We carried out multiple logistic regression analyses of current mobile phone ownership with age, sex, ethnicity, parental socioeconomic classification and type of school. After adjustment for other covariates, we found 62% [odds ratio (OR) = 1.62, 95% confidence interval (CI) 1.34-1.96] higher odds of owning a mobile phone for each year of age. Black, Asian and Mixed ethnicities were associated with lower odds of currently owning a mobile phone compared with adolescents of White ethnicity. For example, Asian adolescents have 82% (OR = 0.18, 95% CI 0.15-0.22) lower odds of owning a mobile phone than White adolescents (Table 5). Adolescents at state schools had considerably lower odds of owning a mobile phone than those at independent schools (OR = 0.40, 95% CI 0.31-0.52). Finally, adolescents whose parental occupations were classified as ‘Higher managerial, administrative and professional’ had higher odds of owning a mobile phone than adolescents with parents in other occupations, except for ‘Intermediate’ (Table 5).

**Table 5.** Multiple logistic regression analyses of sociodemographic variables with mobile phone ownership

	Unadjusted model		Adjusted model^a^	
Independent variables	OR	95% CI	OR	95% CI
Age (years)^b^	1.32	1.13–1.55	1.62	1.34–1.96
Sex				
Male	1.00	–	1.00	–
Female	0.89	0.78–1.02	0.96	0.83–1.12
Ethnicity				
White	1.00	–	1.00	–
Black	0.47	0.37–0.59	0.58	0.45–0.76
Asian	0.16	0.13–0.19	0.18	0.15–0.22
Mixed	0.51	0.39–0.67	0.56	0.42–0.75
Other/not interpretable	0.35	0.26–0.47	0.40	0.29–0.56
Socioeconomic classification				
Managerial/professional occupations	1.00	–	1.00	–
Intermediate occupations	0.85	0.65–1.12	1.06	0.79–1.42
Small employers and own-account workers	0.50	0.42–0.60	0.72	0.59–0.87
Lower supervisory and technical occupations	0.42	0.31–0.56	0.72	0.52–0.98
Semi-routine/routine occupations	0.45	0.37–0.56	0.62	0.50–0.78
Type of school				
Independent	1.00	–	1.00	–
State	0.26	0.21–0.33	0.40	0.31–0.52

*N* = 5539.

The socioeconomic classification is based on the highest National Statistics Socioeconomic Classification (NS-SEC) level (five-group version) of either parent.

a Adjusted for all other independent variables in the table.

b Odds ratios for age indicate the expected increase in odds of owning a mobile phone with a 1-year increase in age.

**Table 6.** Ordinal logistic regression analyses with sociodemographic variables of self-reported mobile phone calls: [(a) frequency (no. of calls), (b) duration (minutes)]

Independent variables	Unadjusted model				Adjusted model^a^			
	Weekday		Weekend		Weekday		Weekend	
	OR	95% CI	OR	95% CI	OR	95% CI	OR	95% CI
(a) Frequency								
Age^b^	1.99	1.77–2.23	1.81	1.61–2.04	1.70	1.49–1.94	1.58	1.39–1.80
Sex								
Male	1.00	–	1.00	–	1.00	–	1.00	–
Female	1.07	0.98–1.18	0.98	0.89–1.07	1.16	1.05–1.28	1.03	0.93–1.15
Ethnicity								
White	1.00	–	1.00	–	1.00	–	1.00	–
Black	2.11	1.83–2.43	2.08	1.80–2.39	1.80	1.54–2.11	1.70	1.45–1.99
Asian	0.80	0.70–0.91	0.73	0.64–0.83	0.65	0.56–0.74	0.60	0.52–0.69
Mixed	1.59	1.35–1.87	1.47	1.25–1.73	1.45	1.22–1.73	1.28	1.07–1.52
Other/not interpretable	1.61	1.30–2.00	1.48	1.20–1.83	1.35	1.06–1.72	1.32	1.04–1.67
Socioeconomic classification								
Managerial/professional occupations	1.00	–	1.00	–	1.00	–	1.00	–
Intermediate occupations	1.14	0.95–1.37	1.09	0.91–1.31	0.97	0.80–1.17	0.94	0.78–1.13
Small employers and own-account workers	1.33	1.14–1.54	1.30	1.12–1.50	1.03	0.88–1.21	1.03	0.88–1.20
Lower supervisory and technical occupations	1.20	0.93–1.54	1.04	0.81–1.34	0.99	0.76–1.28	0.88	0.68–1.14
Semi-routine occupations	1.34	1.13–1.58	1.37	1.16–1.61	1.04	0.88–1.24	1.07	0.90–1.27
Type of school								
Independent	1.00	–	1.00	–	1.00	–	1.00	–
State	2.48	2.23–2.76	2.35	2.11–2.61	2.08	1.83–2.37	2.08	1.83–2.37
(b) Duration								
Age^b^	1.26	1.11–1.42	1.27	1.13–1.42	1.13	0.99–1.29	1.14	1.00–1.29
Sex								
Male	1.00	–	1.00	–	1.00	–	1.00	–
Female	1.31	1.18–1.44	1.27	1.16–1.40	1.37	1.23–1.52	1.36	1.23–1.51
Ethnicity								
White	1.00	–	1.00	–	1.00	–	1.00	–
Black	1.88	1.63–2.17	2.08	1.80–2.40	1.61	1.37–1.89	1.80	1.53–2.11
Asian	0.72	0.63–0.82	0.78	0.69–0.88	0.62	0.54–0.72	0.68	0.59–0.78
Mixed	1.37	1.16–1.62	1.45	1.23–1.70	1.18	0.98-1.41	1.25	1.05–1.49
Other/not interpretable	1.42	1.14–1.77	1.30	1.05–1.62	1.12	0.87–1.43	1.09	0.86–1.38
Socioeconomic classification								
Managerial/professional occupations	1.00	–	1.00	–	1.00	–	1.00	–
Intermediate occupations	1.24	1.02–1.50	1.23	1.02–1.48	1.06	0.87–1.30	1.08	0.89–1.30
Small employers and own-account workers	1.28	1.10–1.49	1.27	1.10–1.48	1.08	0.92–1.27	1.07	0.92–1.26
Lower supervisory and technical occupations	1.18	0.91–1.52	1.21	0.94–1.55	1.06	0.81–1.38	1.06	0.82–1.37
Semi-routine occupations	1.50	1.27–1.78	1.44	1.22–1.70	1.25	1.05–1.50	1.17	0.98–1.39
Type of school								
Independent	1.00	–	1.00	–	1.00	–	1.00	–
State	1.94	1.73–2.17	1.94	1.74–2.17	1.80	1.57–2.06	1.82	1.59–2.07

*N *= 4629.

Odds ratio, indicates changes in odds of being in higher mobile phone call (a) frequency and (b) duration categories associated with the independent variable group relative to the reference group. The socioeconomic classification is based on the highest National Statistics Socioeconomic Classification (NS-SEC) level (five-group version) of either parent.

a Adjusted for all other independent variables in the table.

b Odds ratios for age indicate proportional odds ratios for a 1-year increase in age on level of call (a) frequency and (b) duration [e.g. for each 1-year increase in age, the odds of being in higher mobile phone call (a) frequency or (b) duration categories (see Table 4) on weekdays increase by (a) 99% and 70% and (b) 26% and 13% for the unadjusted and adjusted models, respectively].

### Variables associated with mobile phone call frequency and duration

We performed ordinal logistic regression analyses of self-reported mobile phone call frequency and duration, both on weekdays and on weekends, in relation to covariates. At higher ages, adolescents reported more frequent mobile phone calls, both on weekdays (OR = 1.70, 95% CI 1.49–1.94) and on weekends (OR = 1.58, 95% CI 1.39–1.80), and slightly longer call duration on both weekdays (OR = 1.13, 95% CI 0.99-1.29) and weekends (OR = 1.14, 95% CI 1.00–1.29). Female adolescents self-reported more mobile phone use than males except for call frequency on weekends, with ORs ranging from 1.16, 95% CI 1.05–1.28, for weekday call frequency to 1.37, 95% CI 1.23–1.52, for weekday call duration. Compared with pupils of White ethnicity, adolescents of Black or Mixed ethnicity tended to report higher levels of mobile phone call frequency and duration, whereas pupils of Asian background reported lower call frequency and duration (Table 6a and b). In most analyses, mobile phone call frequency or duration were unrelated to parental socioeconomic classification. However, pupils at state schools reported higher levels of mobile phone use than pupils at independent schools (Table 6a and b).

We have recently published a paper examining the validity of self-reported mobile phone use in SCAMP when compared with mobile operator traffic data.^30^ The findings show that self-reported usage distinguishes between high and low use.

## What are the main strengths and weaknesses?

### Strengths

SCAMP is by far the largest study in the world to prospectively investigate adolescents’ cognitive, behavioural, educational, physical and mental health outcomes in relation to use of mobile phones and other wireless devices (previous studies^31–33^ included fewer than 1000 participants). It is also the largest collection of cognitive data in adolescents in terms of both the variety of different cognitive tasks/domains studied and the number of adolescents for whom the data are available. The cohort includes adolescents from both state and independent schools and a wide range of socioeconomic and ethnic groups.

For approximately 20% of the cohort we have received parental consent to link the school assessment data with traffic data from network operators, medical records, educational records and other routinely collected data, with potential for long-term follow-up.

Additionally, the collection of data on other environmental exposures (e.g. air pollution, noise, green space use) will generate a rich dataset beyond RF-EMF exposures, which will allow for research on a wide range of other environmental and health issues in this important age group.

### Weaknesses

There could be potential for participation bias, with respect to: (i) the schools participating in SCAMP; (ii) the schools who agreed to take part in SCAMP Bio-Zone; (iii) the pupils for whom parental consent for data linkage has been provided; and (iv) the recruitment for the personal environmental exposure monitoring study. However, we have shown that SCAMP findings will be quite widely generalizable, given that the sociodemographic characteristics of the SCAMP cohort are fairly well representative of the target Greater London school population, with respect to school type and the proportion of males to females and White adolescents versus ethnic minorities. The SCAMP cohort is proportionately more affluent than the target population; however, this is common to many cohorts (e.g.^34^^,^^35^). We did not find any appreciable differences in sociodemographic characteristics between the main SCAMP cohort and SCAMP Bio-Zone. In addition, longitudinal analyses should be relatively unaffected by selective participation.

## Where can I find out more? Can I get hold of the data?

Further details about the study are available at [www.scampstudy.org]. Enquiries regarding data access or potential collaboration for research purposes should be sent to SCAMP Principal Investigator (PI) Dr Mireille B Toledano [m.toledano@imperial.ac.uk]. These requests will be considered by the SCAMP Data Access Committee and may require additional ethical approval. (All STATA code available on request.)

**Profile in a nutshell**

- SCAMP is a prospective secondary school-based cohort study investigating whether the use of mobile phones and other wireless devices is associated with cognitive, behavioural, educational, physical and mental health outcomes.
- A total of 6905 pupils (11-15 years of age) take part in an assessment during school time when they are in Year 7 (baseline) and again when they are in Year 9/10 (follow-up).
- Participants are from 39 secondary schools (26 state, 13 independent) in and around Greater London, UK.
- Pupils complete a series of self-report questionnaires on their lifestyle, mood and use of mobile phones and other wireless devices, as well as a cognitive test battery covering non-verbal fluid intelligence, speech processing, cognitive flexibility, sustained attention, inhibition, working memory, visual attention and mental rotation. These data are complemented by optional additional lifestyle questionnaires that pupils and their parents are encouraged to complete at home.
- Furthermore, pupils at 12/39 schools provide non-invasive biological samples (urine/saliva) and anthropometric measurements (SCAMP Bio-Zone). Another subset of the cohort (*n* ∼ 200) participate in a personal and home environmental monitoring study to gather an in-depth understanding of personal environmental exposures.
- Parental consent is requested for linkage of adolescents’ school assessment data to routinely collected data including health and educational records as well as mobile phone traffic data from network operators. For further information see [www.scampstudy.org] or contact the PI, Dr Mireille B Toledano.

## Funding

This work was supported by the UK Department of Health and Social Care via the Research Initiative on Health and Mobile Telecommunications (RIHMT) (grant number: 091/0212), an independent programme of research that is jointly funded by the UK Health Departments, the Medical Research Council, the Health and Safety Executive and industry funders [Vodafone, Arqiva, Carphone Warehouse, BT, 3UK, Everything Everywhere EE (Orange and T-Mobile) and Telefonica Europe Plc (O2)]. The RIHMT is managed by the UK Department of Health and Social Care’s Policy Research Programme [http://www.prp-ccf.org.uk/]. The noise and air pollution enhancements are supported by the National Institute for Health Research Health Protection Research Unit (NIHR HPRU) in Health Impact of Environmental Hazards at King’s College and Imperial College London in partnership with Public Health England (PHE) (grant number: HPRU-2012–10141). The noise and Bio-Zone enhancements were also supported, in part, by funds from the MRC-PHE Centre for Environment and Health (MR/L01341X/1). The views expressed in this publication are those of the authors and not necessarily those of the National Health Service, the NIHR, the Department of Health and Social Care or PHE. P.E. is supported by the Imperial College Healthcare NHS Trust (ICHNT) and Imperial College Biomedical Research Centre (BRC), the MRC-PHE Centre for Environment and Health, the National Institute for Health Research (NIHR) Health Protection Research Unit in Health Impact of Environmental Hazards and the UK Dementia Research Institute (DRI) which receives its funding from UK DRI Ltd funded by the UK Medical Research Council, Alzheimer’s Society and Alzheimer’s Research UK.

## Supplementary Material

Supplementary Tables
