# Supplementary material for: Cohort Profile: The Study of Cognition, Adolescents and Mobile Phones (SCAMP)
Source: Int J Epidemiol. 2018 Oct 15;48(1):25–26l. doi: 10.1093/ije/dyy192 (PMC6380299; doi:10.1093/ije/dyy192)
Supplement: Supplementary Tables [file dyy192_supplementary_tables.docx]

| Supplementary Table 1. SCAMP data collection (personal monitoring, consent form, debrief). | | |
| --- | --- | --- |
| **Personal monitoring questionnaire** | **Questionnaire with consent forms (parent-completed)** | **Online debrief after school assessment** |
| *Mobile phone* | | |
| Current mobile phone ownership | Current mobile phone ownership | School mobile phone policy |
| Type of contract/PAYG | Age first using a mobile phone |  |
| Network operator | Type of contract/PAYG |  |
| Account holder | Network operator |  |
| Frequency/duration of calls | Account holder |  |
| Using other people’s phones |  |  |
| Messaging frequency (text and instant messages) |  |  |
|  |  |  |
| Mobile internet use (incl. proportion using WiFi) |  |  |
|  |  |  |
| Use of hands-free services |  |  |
| Phone disturbance during night |  |  |
| Night-time mobile phone location and settings |  |  |
|  |  |  |
| Location of ExpoM and mobile phone while carrying |  |  |
|  |  |  |
| *Cordless phone* | | |
| Cordless phone ownership |  |  |
| Location of base station |  |  |
| Duration of calls |  |  |
| *Use of other technologies* | | |
| WiFi at home (incl. router location, night-time switching off) |  | School W-LAN policy |
|  |  |  |
| Social networking |  |  |
| Use of other digital technologies |  |  |
| *Device use at school* | | |
|  |  | Desktop computer |
|  |  | Laptop |
|  |  | Tablet |
| *Health and well-being* | | |
| Allergies | Special educational needs |  |
| Disabilities, illness or medical condition | Medication use |  |
|  | Medical condition, incl. age at diagnosis and years affected |  |
|  |  |  |
|  | Treatment for physical or mental health condition |  |
|  |  |  |
| *Pregnancy and child development* | | |
|  | First child |  |
|  | Birthweight |  |
| *Behaviour* | | |
| Play in green spaces | Emotional symptoms, conduct problems, hyperactivity or inattention, peer relationship problems, prosocial behaviour [SDQ]^[24]^ |  |
|  |  |  |
|  |  |  |
|  |  |  |
|  |  |  |
|  | Sport |  |
|  | Vegetarian/vegan |  |
| *Socio-Demographics* | | |
| Age | Age |  |
| Household structure | Sex |  |
| Housing | Height |  |
|  | Weight |  |
|  | Parental age |  |
|  | Parental sex |  |
|  | Parental education |  |
|  | Parental occupation |  |
|  | Parental marital status |  |
|  | Parental ethnicity |  |
|  | Parental birth place |  |
|  | Free school meals |  |
|  | Residential history |  |
| *Environmental factors* | | |
| Slept at own house or elsewhere |  | Quietness of the session |
| Special events during measurement period |  |  |
|  |  |  |
| *Note.* Table shows data that are collected as part of SCAMP’s personal monitoring and online questionnaires as well as data collected during the school assessment for debrief. SDQ = Strengths and Difficulties Questionnaire^[24]^. | | |

| Supplementary Table 2. Baseline sociodemographic characteristics of the SCAMP Bio-Zone cohort^1^. | | | | | | |
| --- | --- | --- | --- | --- | --- | --- |
|  | Overall | | Male | | Female | |
|  | (*N* = 1,981) | | (*n* = 940, 47.45%) | | (*n* = 1,041, 52.55%) | |
|  | Median | IQR | Median | IQR | Median | IQR |
| Age (years)^2^ | 12.18 | 11.92-12.44 | 12.18 | 11.90-12.43 | 12.17 | 11.93-12.46 |
| Ethnicity | *n* | % | *n* | % | *n* | % |
| White | 872 | 44.02 | 488 | 51.91 | 384 | 36.89 |
| Black | 238 | 12.01 | 79 | 8.40 | 159 | 15.27 |
| Asian | 526 | 26.55 | 225 | 23.94 | 301 | 28.91 |
| Mixed | 194 | 9.79 | 88 | 9.36 | 106 | 10.18 |
| Other/Not interpretable | 102 | 5.15 | 34 | 3.62 | 68 | 6.53 |
| Missing | 49 | 2.47 | 26 | 2.77 | 23 | 2.21 |
| Socioeconomic classification |  |  |  |  |  |  |
| Managerial/professional occupations | 1,122 | 56.64 | 582 | 61.91 | 540 | 51.87 |
| Intermediate occupations | 125 | 6.31 | 49 | 5.21 | 76 | 7.30 |
| Small employers and own account workers | 244 | 12.32 | 109 | 11.60 | 135 | 12.97 |
| Lower supervisory and technical occupations | 58 | 2.93 | 24 | 2.55 | 34 | 3.27 |
| Semi-routine routine occupations | 171 | 8.63 | 57 | 6.06 | 114 | 10.95 |
| Missing/Not interpretable | 261 | 13.18 | 119 | 12.66 | 142 | 13.64 |
| Type of school |  |  |  |  |  |  |
| State | 1,369 | 69.11 | 569 | 60.53 | 800 | 76.85 |
| Independent | 612 | 30.89 | 371 | 39.47 | 241 | 23.15 |

*Note.* IQR = interquartile range. The socioeconomic classification is based on the highest National Statistics Socio-economic Classification [NS-SEC] level (five group version) of either parent.

^1^ Data based on participants who took part in the Bio-Zone assessment at baseline and who could be linked to the computer-based assessment at baseline.
